# Endovascular Treatment of Acute Carotid Stent Occlusion: Aspiration Thrombectomy and Angioplasty

**DOI:** 10.7759/cureus.7997

**Published:** 2020-05-06

**Authors:** Nick M Murray, Dylan N Wolman, Michael Marks, Robert Dodd, Huy M. Do, Jason T Lee, Jeremy J Heit

**Affiliations:** 1 Neurology, Stanford University, Stanford, USA; 2 Neuroradiology, Stanford University, Stanford, USA; 3 Neurosurgery/ Cerebrovascular and Skull Base Surgery, Stanford University Medical Center, Palo Alto, USA; 4 Radiology, Stanford University School of Medicine, Stanford, USA; 5 Vascular Surgery, Stanford University, Stanford, USA; 6 Radiology, Stanford University, Stanford, USA

**Keywords:** acute carotid stent occlusion, endovascular treatment, aspiration thrombectomy, angioplasty, acute stroke, neurointerventional radiology

## Abstract

Introduction

Acute carotid stent occlusion (CSO) is a rare complication of endovascular carotid stent placement that requires emergent intervention. We describe angioplasty or combined angioplasty and aspiration thrombectomy as a new endovascular technique for CSO treatment. The technique is compared to others previously described in the literature.

Methods

We performed a retrospective cohort study of all patients who underwent endovascular treatment (ET) of acute symptomatic CSO from January 2008 to March 2018 at our neurovascular referral center. Patient demographics, endovascular treatment details, and outcome data were determined from the electronic medical record. Primary outcome was successful stent recanalization and cerebral reperfusion (modified thrombolysis in cerebral infarction (mTICI) score IIB-III). Secondary outcomes were National Institutes of Health Stroke Scale (NIHSS) shift from presentation to discharge, mortality, and modified Rankin Scale (mRS) score at 3 months. Additionally, a literature review (years 2008-2019) was performed to characterize other techniques for ET of CSO.

Results

Four patients who underwent ET of acute CSO were identified. ET treatment by angioplasty (n = 1) or combined aspiration thrombectomy and angioplasty (n = 3) resulted in carotid stent recanalization in all patients. Tandem intracranial occlusions were present in three patients (75%), and successful cerebral reperfusion was achieved in all patients. Patient symptoms improved (mean NIHSS shift -5.3 ± 7.2 at discharge). One patient died of a symptomatic reperfusion hemorrhage and another died of cardiac complications by 3-month follow-up. The mRS scores of the surviving patients were 1 and 3. Previously described studies (n = 14) using different and varied techniques had moderate recanalization rates and outcomes.

Conclusion

Combined aspiration thrombectomy and angioplasty for the neurointerventional treatment of acute CSO leads to high rates of stent recanalization and cerebral reperfusion. The recanalization rate here is improved compared to previously reported techniques. Further multicenter studies are required to risk-stratify patients for specific ET interventions.

## Introduction

Acute carotid stent occlusion (CSO) is a rare cause of acute ischemic stroke that is associated with significant morbidity and mortality [1]. CSO occurs in 0.05% to 0.8% of patients with the internal carotid artery (ICA) or common carotid artery stents and is caused by antiplatelet medication noncompliance or discontinuation, antiplatelet medication resistance, overlapping stent placement, or intrinsic prothrombotic disorders [1-4]. In addition, procedural events and complications, such as dissection, atheroma perturbation, or ICA kinking after stent placement, may predispose a stent to occlusion [5].

CSO treatments include conservative medical therapy, endovascular treatment (ET), surgical stent explantation, carotid endarterectomy, or a combination of these approaches [1,3,5-7]. ET of CSO presents challenges for neurointerventionalists, and the risk of revascularization techniques must be balanced against the risk of clot propagation, carotid stent damage, and reperfusion injury [3,5]. Described ET techniques for CSO include intra-arterial (IA) thrombolysis often with tissue plasminogen activator (tPA) or glycoprotein IIb/IIIa receptor inhibitors and aspiration thrombectomy with or without mechanical thrombectomy [5,8-12]. Angioplasty has been described for in-stent stenosis and intraprocedural expansion of incompletely secured stents with thrombus formation, but not for CSO [3,5,13-14].

We present the first report of combined angioplasty and aspiration thrombectomy for the treatment of acute CSO. All patients underwent angioplasty to promote thrombus disruption and restore antegrade flow through the occluded carotid stent. Residual in-stent thrombus was removed using aspiration thrombectomy. This technique is described in detail, and its effectiveness is compared to the literature for ET of CSO.

## Materials and methods

Patient information

The study was approved by the Institutional Review Board (IRB) and complied with the Health Insurance Portability and Accountability Act. The need for informed consent was waived the IRB. We retrospectively reviewed our neurointerventional database to identify consecutive patients who underwent ET for acute CSO treatment between January 2008 and March 2018. Patient demographics, endovascular treatment details, and outcome data were determined from the electronic medical record.

Among patients who underwent pre-interventional perfusion imaging, automated post-processing was performed using RApid processing of PerfusIon and Diffusion (RAPID) software (iSchemaView, Menlo Park, CA). Core infarct and penumbral volumes (defined as the volume of tissue with time-to-maximum (Tmax) >6 seconds) were determined using RAPID. Patients had pre-interventional computed tomography angiography (CTA) or magnetic resonance angiography (MRA). In one patient, the CTA was non-diagnostic due to technical issues. In this patient, a virtual CT angiogram was reconstructed from the perfusion source images.

All patients who undergo carotid stent placement at our institution undergo surveillance CTA at 3, 6, and 12 months to evaluate for in-stent stenosis. However, patients who present with CSO before these follow-up appointments were not screened for an in-stent stenosis. Therefore, only one patient in this series underwent follow-up imaging due to delayed presentation (patient four). Antiplatelet resistance in patients with verified medication compliance, and no other identified cause of CSO was verified using thromboelastography, with secondary testing by a hemostasis platelet function assay-100 (PFA-100) system (Siemens, Tarrytown, NY).

Endovascular treatment of CSO

All patients underwent ET in a biplane neuroangiography suite (Axiom Artis, Siemens) under either monitored anesthesia care or general anesthesia. Common femoral artery access was obtained using standard techniques, and an 8- or 9-French sheath was placed in the descending thoracic aorta. Access into the common carotid artery of the affected hemisphere was obtained with a 5-French Berenstein angiographic catheter (Cordis, Milpitas, CA), which was placed through a 6-French shuttle sheath (Cook Medical, Bloomington, IN). Selective digital subtraction angiography (DSA) of the affected common carotid artery was performed prior to ET.

**Figure 1:**
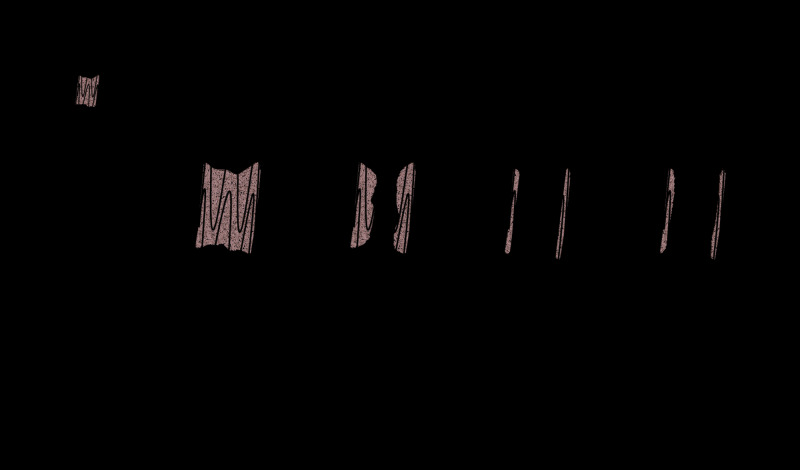
Schematic of endovascular treatment of CSO Schematic for endovascular recanalization of CSO. After obtaining access to the affected common carotid artery (A), an aspiration catheter is advanced through the thrombosed stent under continuous aspiration and removed (B). An embolic protection device is placed distal to the stent, and angioplasty is performed to macerate the residual thrombus against the stent wall (C). Post-angioplasty DSA demonstrates stent recanalization with minimal residual thrombus (D). CSO, carotid stent occlusion; DSA, digital subtraction angiography

The affected common carotid artery was accessed (Figure 1A), and an aspiration catheter was advanced and withdrawn through the thrombosed stent into the more distal cervical ICA under continuous aspiration to at least partially recanalize the CSO (Figure 1B). Next, an embolic protection device (Accunet, Abbott, Abbott Park, IL) was placed distal to the stent over a guidewire (Figure 1C) in three patients. The embolic protection device could not be navigated through the partially occluded stent in the fourth patient. A balloon catheter was then advanced within the partially recanalized stent and step-wise angioplasty was performed to macerate the residual thrombus against the stent wall (Figure 1C). Different balloon catheters were used for each patient: a 4x12mm monorail balloon catheter (Boston Scientific, Marlborough, MA), a non-compliant Trek 5x15mm balloon microcatheter (Abbott, Abbott Park, IL), a Trek rapid exchange 5x12mm balloon catheter (Abbott, Abbott Park, IL), and a Viatrac 6x20mm balloon catheter (Abbott, Abbott Park, IL). Post-angioplasty DSA was performed demonstrating improved stent caliber with minimal residual thrombus (Figure 1D).

Tandem intracranial occlusions were treated with a combination of IA-tPA, aspiration, and mechanical thrombectomy. Detailed ET descriptions for each patient are presented in the Appendix Text.

Technique effect and outcome metrics

The primary outcome was successful CSO recanalization and cerebral vascular reperfusion (modified thrombolysis in cerebral infarction, (mTICI) score IIB-III). Secondary outcome measures were National Institutes of Health Stroke Scale (NIHSS) shift from presentation to discharge, modified Rankin Scale (mRS) score at 3 months and mortality [15-16].

Literature review

**Figure 2:**
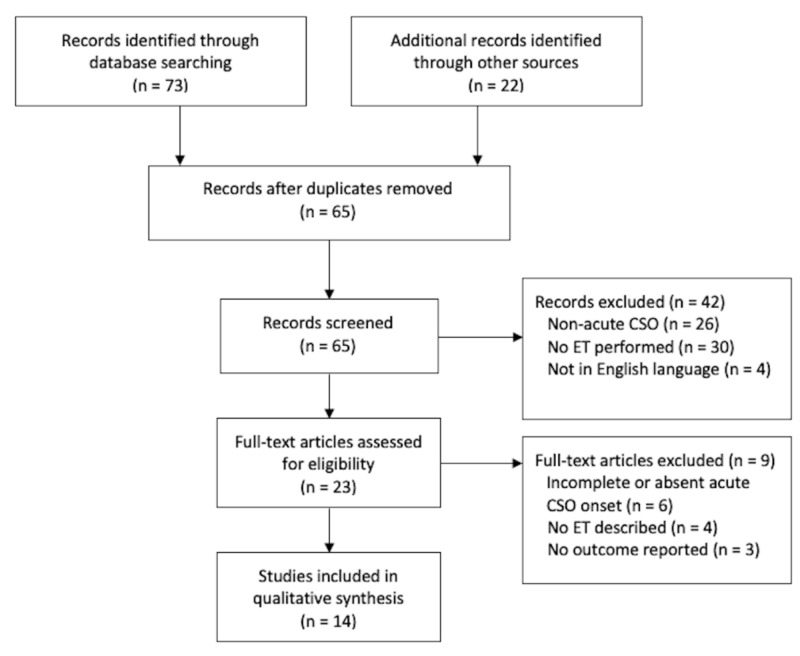
PRISMA flow diagram of database search and literature review PRISMA, preferred reporting items for systematic reviews and meta-analyses; ET, endovascular therapy; CSO, carotid stent occlusion

A comprehensive review of PubMed and Embase was performed to identify all eligible acute CSO treatment studies published since January 2008, with the last update of literature review on July 2019. Search terms used included: “carotid stent” AND “occlusion”, “carotid artery stent” AND “occlusion”, “carotid stent” AND “thrombosis”, “carotid artery stent” AND “thrombosis”, or “carotid” AND “stent” AND “occlusion”. Inclusion criteria were: English language, acute onset of CSO (i.e. presentation of acute stroke symptoms leading to discovery of the CSO) irrespective from time of original stent placement, and publication dates January 2008 - July 2019. In total, 65 non-duplicate studies were identified and 51 were excluded due to: absence of complete CSO, non-acute CSO, no surgical or ET intervention performed or described, incomplete or absent characterization of CSO onset, etiology, and/or outcome (Figure 2). Two authors reviewed each study that met all inclusion criteria and did not meet exclusion criteria. 

## Results

Endovascular treatment of CSO

**Figure 3:**
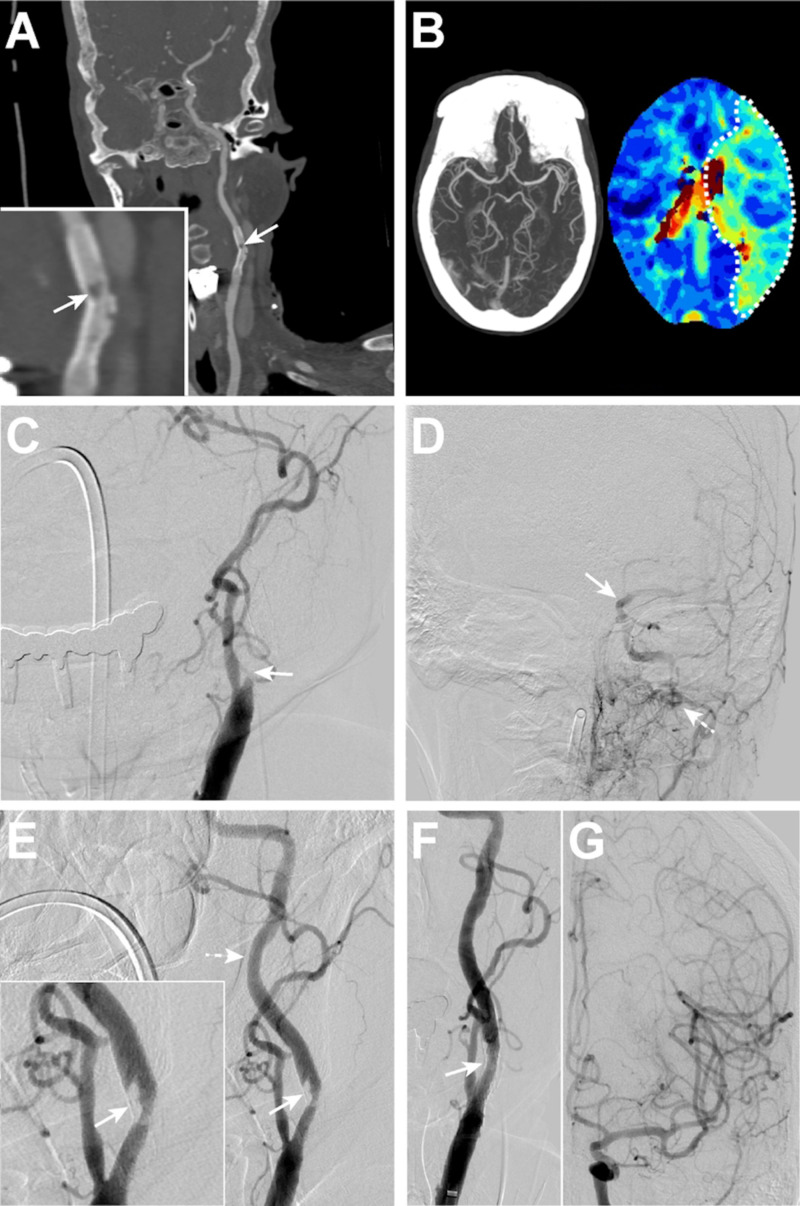
Patient 1 with left CSO seven days post stent placement (A) Coronal maximum-intensity-projection images following a CTA demonstrates a thrombus within the left internal carotid artery stent (arrow). Inset shows magnified region of stent with thrombus (arrow). (B) Left: Maximum intensity projection of the Circle of Willis CTA demonstrates no intracranial large vessel tandem occlusions. Right: CT perfusion imaging shows a perfusion deficit (Tmax >6 seconds) in the left middle cerebral artery territory (dashed outline) secondary to the carotid stent thrombus. (C-G) Left common carotid artery DSA images. There is occlusion of the left carotid stent (c, arrow) with no antegrade filling of the cervical left ICA (c) and poor filling of the intracranial left ICA (d, arrow), largely via left external carotid artery collaterals (d, dashed arrow). (E) CSO treatment by aspiration thrombectomy resulted in antegrade filling of the left ICA stent with residual non-occlusive thrombus within the stent (arrow; inset, arrow) and improved filling of the more distal cervical ICA (dashed arrow). (F) Subsequent angioplasty resulted in minimal residual thrombus within the stent (arrow). (G) Robust antegrade filling of the left anterior circulation was present after CSO treatment. CTA, computed tomography angiography; ICA, internal carotid artery; DSA, digital subtraction angiography; CSO, carotid stent occlusion

**Figure 4:**
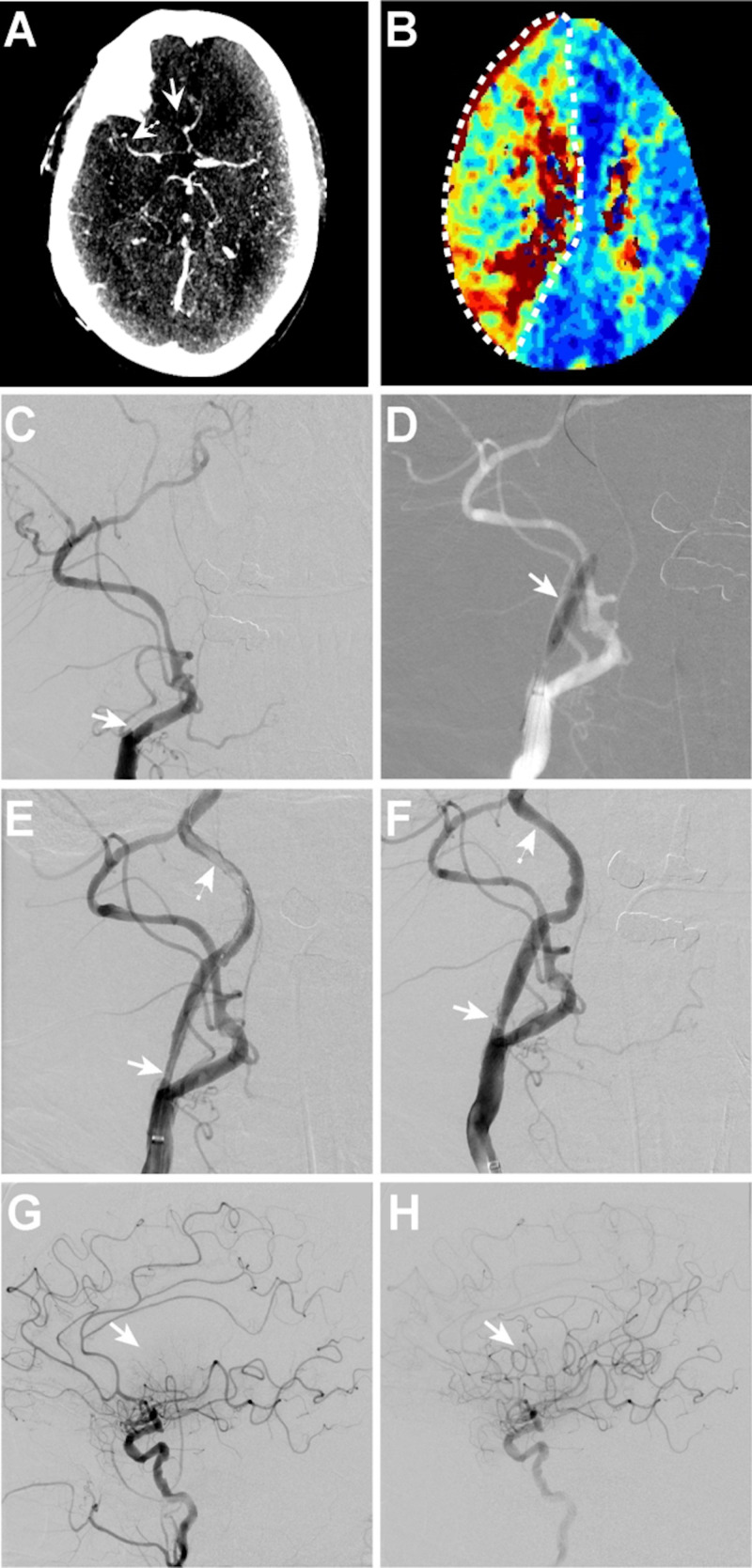
Patient 2 with right CSO eight hours post stent placement with tandem anterior cerebral artery and middle cerebral artery occlusions (A) Due to technical limitations, a CTA was not obtained, thus CT perfusion source data was reformatted into a 5mm MIP from the peak vascular enhancement series using manual bone masking at the skull base to demonstrate the pre-intervention tandem occlusions of the right A2 (arrow) and M2 (dashed arrow) segments of the anterior and middle cerebral arteries. (B) CT perfusion imaging shows a perfusion deficit (Tmax >6 seconds) in the right middle and anterior cerebral artery territory (dashed outline) secondary to the carotid stent thrombus. (C-H) Right common carotid artery DSA images. (C) There is occlusion of the right carotid stent (arrow) with no antegrade filling of the cervical right ICA (arrow). (D) Balloon angioplasty of the right carotid stent (arrow) was performed. (E) After aspiration thrombectomy and angioplasty, there is residual thrombus in the carotid stent (arrow) with additional non-occlusive thrombus in the more distal right ICA (dashed arrow). (F) After additional aspiration thrombectomy, there is minimal residual thrombus in the carotid stent (arrow) and no residual thrombus in the more distal right ICA (dashed arrow). (G) Following CSO treatment, there is poor filling of the right middle cerebral artery inferior divisions (arrow), as well as the A2 segment of the right anterior cerebral artery. (H) After combined cerebral aspiration and mechanical thrombectomy, there is excellent reperfusion of the right anterior circulation (arrow). CT, computed tomography; CTA, computed tomography angiography; ICA, internal carotid artery; DSA, digital subtraction angiography; CSO, carotid stent occlusion

In our institutional neurointerventional database, we identified four patients who underwent ET for symptomatic acute CSO. Patient demographic and treatment data are described in Table 1. The mean presentation NIHSS score was 14.5 ± 3.9. Three patients underwent perfusion imaging prior to treatment, and, in these patients, the mean infarct core was 0.7 ± 0.7 ml and penumbra (Tmax > 6) was 110.5 ± 59.7 ml. Three patients were treated with aspiration thrombectomy and angioplasty, and the fourth underwent angioplasty alone for CSO treatment. Procedural details are described in the above Materials and Methods section, with full technical treatment details for each patient described in the Appendix Text. Successful CSO recanalization was achieved in all four patients (100%) (Figures 3 & 4; Appendix Figures 5 & 6). Tandem intracranial occlusions were present in three patients, and two of these patients had evidence of these occlusions on pre-ET imaging (Figure 3, Appendix Figure 6). All intracranial occlusions were successfully revascularized with mTICI IIB-III reperfusion (100%; Table 1).

**Table 1:** Acute carotid stent occlusion patient clinical characteristics, stent information, and outcomes after endovascular therapy (ET). ET for all patients was aspiration thrombectomy, followed by angioplasty, except patient 4 who was treated with angioplasty only. ET, endovascular treatment; CSO, carotid stent occlusion; NIHSS, National Institutes of Health Stroke Scale; mTICI, modified thrombolysis in cerebral infarction; ICA, internal carotid artery; HTN, hypertension; DM, diabetes mellitus; HLD, hyperlipidemia; CAD, coronary artery disease; OSA, obstructive sleep apnea; MI, myocardial infarction; PVD, peripheral vascular disease; DAPT, dual antiplatelet; SAPT, single antiplatelet; IA-tPA, intra-arterial tissue plasminogen activator.

Patient	Past Medical History	Carotid Stent Side	Pre-Stenting Stenosis	Pre-Stenting Anti-thrombotic(s)	Stent Type	Post-Stent & Pre-ET NIHSS	Latency to Acute Thrombosis (CSO)	Anti-thrombotic Regimen at time of CSO	CSO Etiology	Symptoms & NIHSS at Presentation	Time from last known normal	mTICI Score / Tandem Treatment	Post ET NIHSS	Mortality at 3 months	mRS at 3 months
1	Tonsil cancer with resection and x-ray therapy, CAD, HTN, HLD, Hypothyroidism, DM	Left	90% of distal bulb and proximal ICA	Aspirin 81 mg, Clopidogrel 75 mg	Acculink carotid stent system 6-8 mm taper x 30 mm	0	7 days	Aspirin 81 mg, Clopidogrel 75 mg	Clopidogrel resistance, inconsistent clopidogrel compliance, radiation vasculopathy	Right arm hemiparesis, NIHSS 4	0.5 hr	III No tandem occlusions	0	Alive	1
2	Atrial fibrillation, CAD, HTN, HLD, OSA, bilateral carotid stenosis with remote carotid endarterectomy	Right	> 90% proximal ICA	Clopidogrel 300 mg Coumadin, INR at 2-3	Xact stent 9 x 40 mm	0	8 hours	Protamine 100 mg, Clopidogrel 75 mg	Protamine reversal after heparin in the setting of atrial fibrillation	Left hemiparesis, NIHSS 13	0.25 hr	IIB Aspiration thrombectomy, Solitaire Retriever	3	Alive	3
3	CAD with MI, HLD, DM, smoker, PVD, chronic right ICA occlusion	Left	80% ICA	Aspirin 81 mg, Clopidogrel 75 mg	Xact stent 8 x 40 mm	0	6 days	Aspirin 81 mg, Clopidogrel 75 mg	Noncompliance with DAPT (not taking any antiplatelet)	Aphasia and right hemiparesis, NIHSS 20	12 hr	IIB IA-tPA	18	Deceased	6
4	CAD with MI	Left	>70% of ICA reconstruction	Clopidogrel 75 mg	Xact stent 9 x 30 mm	0	18 months	Aspirin 325 mg, Clopidogrel 75 mg	Noncompliance with DAPT (not taking any antiplatelet)	Aphasia and right hemiparesis, NIHSS 21	7 hr	IIB Merci Retriever	42	Deceased	6

Following ET, one patient expired after a cerebral reperfusion hemorrhage. At discharge, the mean NIHSS shift in the remaining three patients was -5.3 ± 7.2­. By three months after ET, one additional patient had expired from a myocardial infarction, and the two surviving patients had 3-month mRS scores of 1 and 3 (Table 1).

Literature review of acute CSO treatment

Acute CSO is most commonly secondary to antiplatelet resistance, intolerance, or noncompliance (Table 2). Previously described ET techniques for CSO treatment apply methods used for non-stented carotid occlusions, and include IA thrombolysis, IA aspiration, heparinization, and mechanical thrombectomy [5,8-12,14,17-24]. A total of 23 patients were described, with most achieving recanalization (approximately 85-90%). However, outcomes reported range from a general single classification of either improved or not improved, or a spectrum of significant new functional deficits (Table 2). The nonuniform reporting of TICI score, mRS, and mortality data limits precise comparative analytics between the studies and their techniques. 

**Table 2:** Literature review of acute carotid stent occlusion etiology, treatment, and outcome (2008-2019) Twenty-three patients were described; however, the success rates of ET as measured by mTICI score nor mRS are not uniformly reported. CSO, carotid stent occlusion; IA, intraarterial; IV, intravenous; tPA, tissue plasminogen activator; CEA, carotid endarterectomy; ET, endovascular treatment; mTICI, modified thrombolysis in cerebral infarction; STA-MCA, superficial temporal artery-middle cerebral artery; DAPT, dual antiplatelet; DSA, digital subtraction angiography; mRS, modified Rankin score [5], [11-12], [14], [19-27]

Author	Year	N	Time to Acute CSO (patients separated by “,”)	CSO Etiology	Treatment Method	mTICI Score	Outcome	Tandem Occlusion
Toljan et al.	2019	1	2 hours	Clopidogrel resistance	Aspiration thrombectomy, IA-tPA, IV eptifibatide	-	Thrombus resolved, deficits not described at 3 months	None
Hu et al.	2018	1	2 minutes	Incomplete stent adherence poor expansion, baseline stenosis remained	Heparin, Intrathrombus tPA, salvage angioplasty to re-expand stent	-	Thrombus resolved, no deficits	Not reported
Moulakakis et al.	2018	2	30 minutes, 1 hour	Plaque protrusion through sent, not reported	Surgical stent explantation, IA tPA, then surgical stent explantation	-	Complete resolution of symptoms in both patients	Not reported
Moulakakis et al.	2017	4	1 hour, 2 hours, 3 days, 4 days	Dissection, two overlapping stents, two overlapping stents and malignancy, two overlapping stents	Aspiration thrombectomy with CEA and stent explantation, IA urokinase and aspiration thrombectomy with additional stent placement, Tinzaparin, Nadroparin with Aspirin and Clopidogrel	-	Mechanical ET with full recanalization, IV only treatment with no recanalization, all with mild residual hemiparesis or speech impairment	Not reported
Koklu et al.	2015	1	1 day	Aspirin and Clopidogrel resistance	Ticlopidine, Heparin	-	Improved dysarthria, stable right hemiplegia	Not reported
Munich et al.	2014	1	Intraprocedural	Embolic protection device thrombus	Aspiration with penumbra 4Max	-	25-30% residual stenosis, no new deficits	Not reported
Kim et al.	2013	3	Intraprocedural (all)	Embolic protection device thrombus	Forced arterial suction thrombectomy with Penumbra	III	All improved	Not reported, no indication intracranial thrombectomy was required and all TICI 3
Kanemaru et al.	2013	1	6 days	Hypercoagulability of malignancy	Aspirin, Clopidogrel, Cilostazol, Coumadin	-	Thrombus resolved, no new deficit	Not reported, no DSA
Markatis et al.	2012	1	2 days	Noncompliance with DAPT	Heparin, surgical exploration, thrombectomy with stent removal	-	Sensory loss on right hand	Not reported
Choi et al.	2012	2	4, 9 days	Not known, Aspirin and Clopidogrel resistance	STA-MCA anastomosis	-	Improved hemiparesis and dysarthria improved	
Iancu et al.	2010	2	Intraprocedural	Carotid dissection, balloon rupture	Intrathrombus Streptokinase, Intrathrombus Tenecteplase and stent secured and expanded with angioplasty	-	Thrombus resolved, no new neurological deficits	Not reported
Naito et al.	2010	2	2 months, 7 days	DAPT discontinued for surgery and hypercoagulability of malignancy, noncompliance	Aspiration thrombectomy, Aspiration thrombectomy and Urokinase		Thrombus resolved, no noted new deficit	Not reported
Dhall et al.	2010	1	Intraprocedural	-	IA Urokinase and Abciximab, aspiration thrombectomy	-	Thrombus resolved, no new neurological deficit	Not reported
Seo et al.	2008	1	Intraprocedural	Distal stent filling defect	IV Tirofiban	III	Complete recanalization	

## Discussion

We describe a novel method of effective ET for symptomatic CSO using a combined aspiration thrombectomy and angioplasty technique. Our technique resulted in the successful recanalization of the CSO in 100% of patients described here and has become the standard intervention for CSO at our institution.

The etiology of CSO in our series was due to inadequate medical platelet inhibition secondary to poor medication compliance, intrinsic antiplatelet resistance, or unintentional anticoagulation reversal, which is similar to prior studies [1-4]. These etiologies most likely led to acute in-stent thrombus formation, which was amenable treatment using our technique. Furthermore, the variable timing of CSO in our series suggests that even carotid stents that are likely well endothelialized are at risk of CSO in the absence of adequate antiplatelet or anticoagulation protection.

Symptomatic acute CSO necessitates emergent intervention regardless of the interval between initial stent placement and presentation as recanalization is the single modifiable outcome predictor for carotid occlusions [18,28]. ET techniques for CSO treatment include IA-tPA, aspiration, and mechanical thrombectomy and remain the most common approach for the treatment of CSO [5,8-12,14,19-24,26]. These techniques result in CSO recanalization in most, but not all (approximately 85-90%), of cases (Table 2), which is lower than the 100% efficacy of our technique. The nonuniform reporting of mTICI score, mRS, and mortality limits outcome comparison between studies using different ET techniques. 

Aspiration thrombectomy for CSO has been described as an effective treatment [12,23]. However, we found aspiration thrombectomy alone resulted in insufficient CSO recanalization in all three patients who underwent this technique before angioplasty. In our cohort, aspiration thrombectomy created a channel through the in-stent thrombus that allowed for the passage of a balloon catheter for subsequent angioplasty.

Combining other ET techniques with aspiration thrombectomy may have a good effect on full recanalization. Angioplasty for acute CSO has not been described, and we have applied this technique as an additional revascularization technique. Angioplasty is often reserved for placing and re-expanding stents that were not already fully secured and were associated with a thrombus [5,14]. Alternative techniques that combine mechanical and aspiration thrombectomy for CSO have been described as similarly effective [10-11]. However, pulling a stent retriever through a recently placed, non-endothelialized carotid stent may risk stent retriever detachment from the pusher wire.

An in-stent stenosis secondary to intimal hyperplasia may predispose to CSO. However, in our series, there was an easy passage of the balloon microcatheter and aspiration catheter through the stent and unremarkable serial surveillance CTA and CT perfusion scans, which suggests an absence of significant in-stent stenosis. In 75% of patients in our series, an embolic protection device was deployed to reduce the theoretical risk of secondary thromboembolism during angioplasty, though no debris was observed within the embolic protection devices at procedure end. Protection against emboli from the stent may be obtained with the use of a balloon-guide catheter, although these catheters are not routinely used at our institution.

Surgical management of CSO by stent explantation, carotid endarterectomy, or carotid artery bypass grafting is considered an alternative in patients with verified dual anti-platelet resistance or as a rescue procedure for failed ET [6,22]. As endovascular techniques continue to improve, we anticipate that the need for surgical treatment of CSO will be further reduced.

The frequency of tandem intracranial occlusions in the setting of CSO remains poorly described, and we found tandem occlusions in 3 of 4 of our patients. Of these patients, 2 had evidence of these occlusions prior to ET, and the third patient developed new tandem occlusions within the A2 segment of the left anterior cerebral artery and distal M2 segment of the left middle cerebral artery despite the use of a distal embolic protection device (Appendix Figure 4). This new embolic occlusion may have occurred prior to, during placement of the embolic protection device, or following angioplasty. In the absence of frequent interval intracranial imaging during ET, it is challenging to conclude if a component of the ET itself was causal for the distal emboli.

Tandem intracranial occlusions were successfully revascularized in all patients in our series using standard endovascular techniques [18,28]. Of note, IA-tPA is most commonly used for acute occlusions at the time of stent placement, for which it is an effective treatment [9]. IA-tPA was used in two patients in our series beyond six hours' time since last seen normal. The clinical benefit of IA-tPA for CSO treatment before or after six hours since last seen normal remains unclear, as in other types of stroke from large vessel occlusion in the form of carotid occlusion, it may reduce mortality but not change functional outcome [5,17-18].

A single patient in our series experienced a fatal reperfusion hemorrhage, which is a risk inherent to all endovascular reperfusion therapies. Our series did note a high 50% mortality rate at 3 months, which partially reflects both the symptomatic nature of the CSO and the severe medical co-morbidities of our cohort. The odds of a poor outcome or death would likely be higher if the recanalization of the occluded stents was not achieved. Non-intervention for CSO has not been studied, but it is likely to result in poor outcomes; comparison to patients who do not undergo thrombectomy for large vessel occlusions supports this hypothesis [29]. A recent systematic literature review found in nearly 60% of patients treated for CSO, that there was either no improvement after therapy or outcomes were not reported [30]. The same is true for nonuniform reporting of outcomes in the literature reviewed here. Notably, some studies that reported more favorable outcomes characterized patients with an asymptomatic CSO. 

No procedural complications or damage to the previously placed carotid stents occurred in our series. A meta-analysis or multicenter experience will likely be required to definitively describe the procedural risk of ET for CSO given the rarity of this event.

Limitations

Our study is limited by its retrospective design, single-center experience, and small sample size. Despite these limitations, this series is, to our knowledge, the largest reported series of effective endovascular treatment of CSO. While variability in the time of presentation, and likely varying degrees of stent endothelization exist within our cohort, it is representative of the spectrum of onset for CSO. We find that the heterogeneity in presentation reflects the reality of CSO occurrence and increases the generalizability of our findings to most patients with an acute CSO.

## Conclusions

Combined aspiration thrombectomy and angioplasty is a viable technique for the neurointerventional treatment of CSO and results in high rates of stent recanalization and cerebral reperfusion, which are both improved from that of previous techniques. Patient symptoms as measured by mean NIHSS shift also improved; however, the severe non-neurological comorbidities within our cohort led to high mortality by 3-month follow-up. Further multicenter studies are required to risk-stratify patients for specific ET interventions.

## Figures and Tables

**Figure 5:**
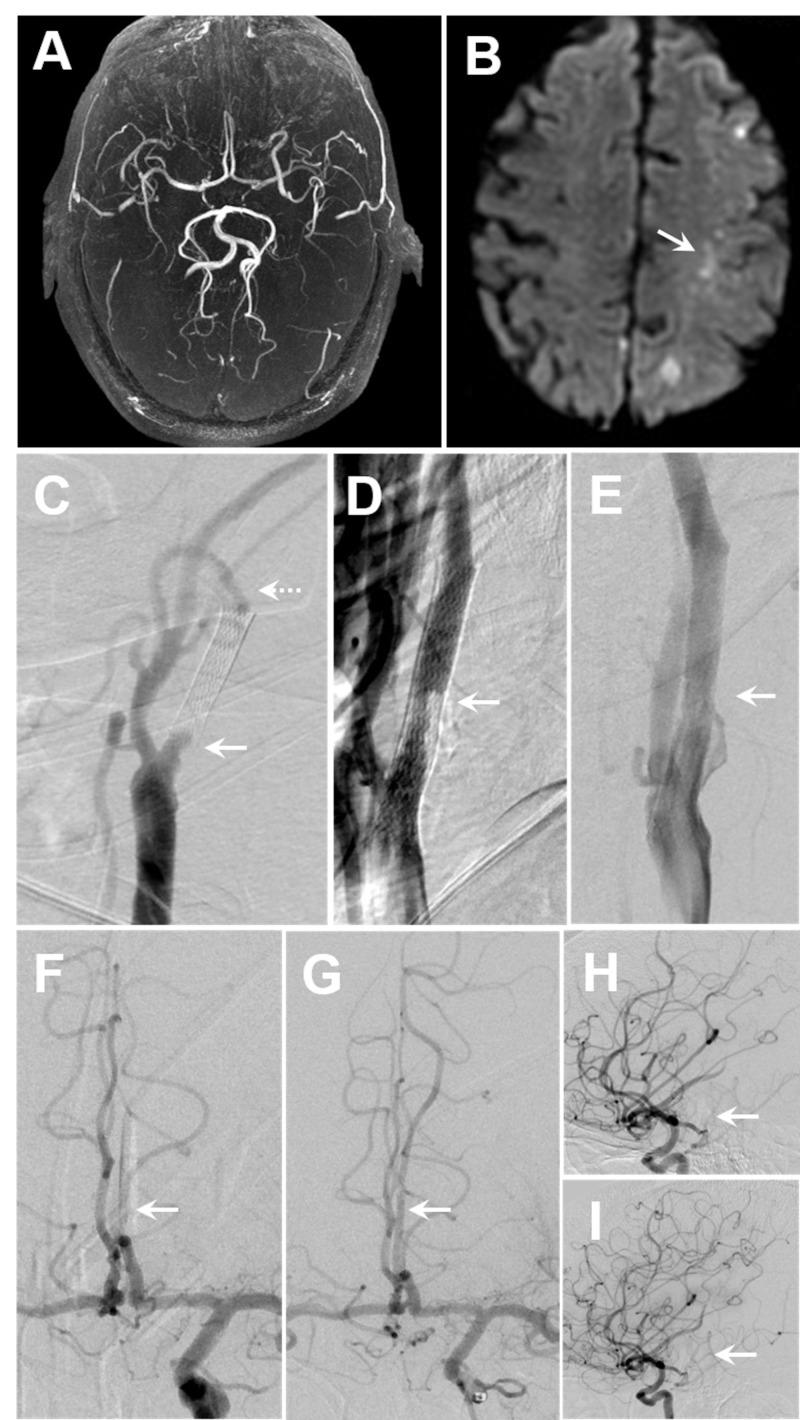
Left CSO 6 days post stent placement with tandem middle cerebral artery M2 segment and anterior cerebral artery A2 segment occlusions (A) Maximum intensity projection of the Circle of Willis MRA demonstrates filling of the intracerebral vasculature with relative paucity of left MCA vessels, and absence of bilateral internal carotid flow. The right carotid was known to be chronically occluded.  (B) MRI diffusion restriction shows a small burden of left ACA/MCA watershed ischemia (arrow). (C-I) Left common carotid artery and intracranial arterial DSA images. (C) There is occlusion of left carotid stent (arrow) with no antegrade filling of the cervical ICA (dashed arrow).  (D) Aspiration thrombectomy of the left carotid stent, with residual non-occlusive thrombus that was treated with balloon angioplasty.  (E) Following additional balloon angioplasty, there was no residual thrombus in the stent (arrow). Intracranial vasculature: (F & H) following aspiration thrombectomy, before carotid stent angioplasty, anterograde flow was observed and new tandem occlusions of the (F) left A2 segment of the ACA and (H) M2 segment of the MCA (arrows) were treated with IA-tPA. This resulted in mTICI IIB reperfusion of the (G) A2 segment of the ACA and (I) M2 segment of the MCA (recanalization marked by arrows). MRA, magnetic resonance angiogram; MCA, middle cerebral artery; MRI, magnetic resonance imaging; ACA, anterior cerebral artery; DSA, digital subtraction angiography; ICA, internal carotid artery; IA-tPA, intra-arterial tissue plasminogen activator; mTICI, modified thrombolysis in cerebral infarction.

**Figure 6:**
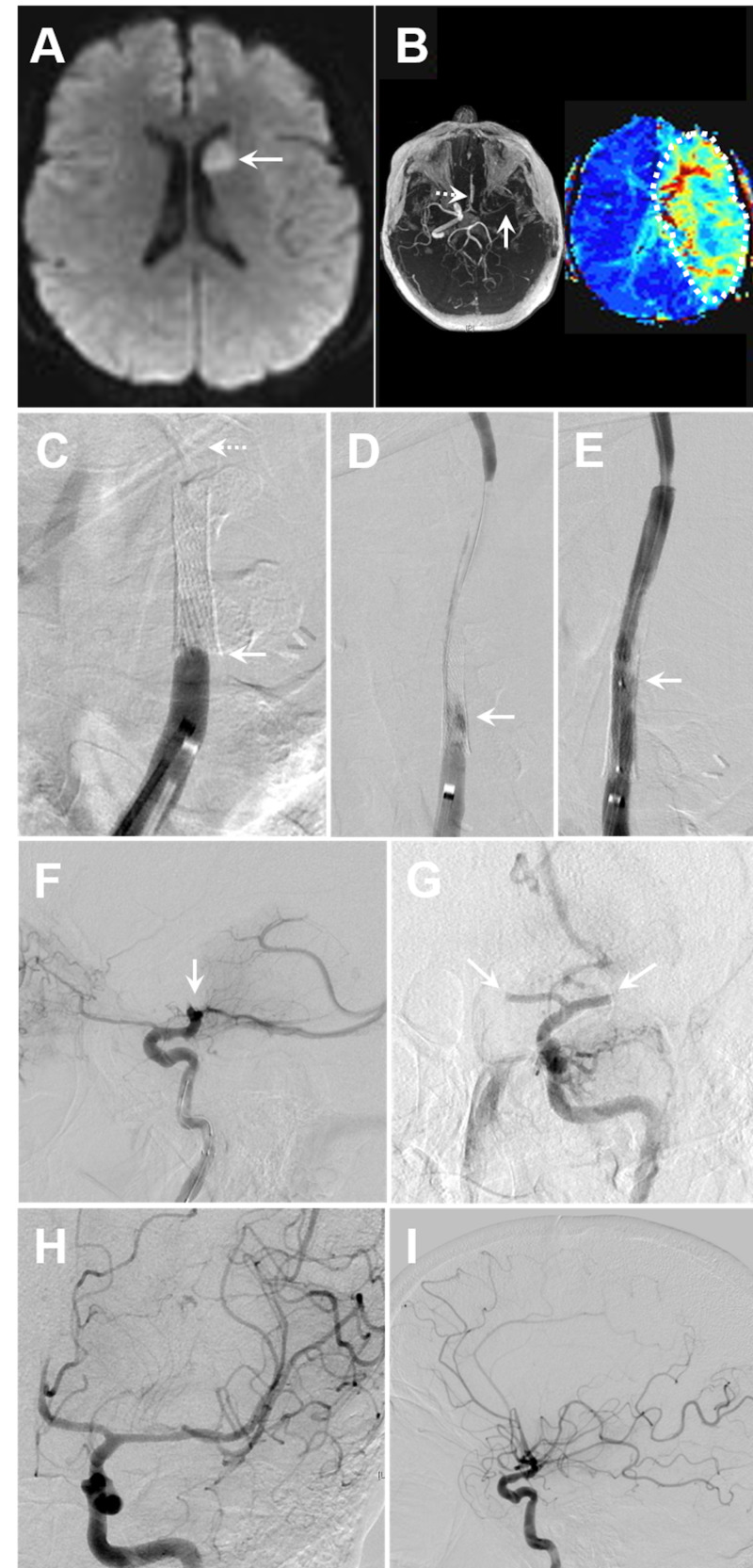
Left CSO 18 months post stent placement with tandem middle cerebral artery M1 segment and anterior cerebral artery A1 segment occlusions (A) MRI showing diffusion restriction of the left caudate head.  (B) Left: maximum intensity projection of the Circle of Willis MRA demonstrates occlusion of the left internal carotid artery and tandem proximal left M1 (arrow) and A1 (dashed arrow) segment occlusions. Right: MRI perfusion imaging shows a large perfusion deficit (Tmax >6 seconds) of the left MCA territory (dashed outline) secondary to carotid stent thrombosis. (C-I) Common carotid to intracranial vasculature DSA images.  (C) There is occlusion of the left carotid stent (arrow) with no anterograde filling of the cervical left ICA (dashed arrow). (D) Angioplasty for CSO treatment resulted in (E) anterograde filling of the left ICA stent an additional angioplasty was performed for complete recanalization.  Following CSO treatment the (F) tandem occlusion of the carotid terminus (sagittal DSA, arrow), was recanalized after IA-tPA.  (G) Pre-ET tandem A1 and M2 tandem occlusions then remained (DSA coronal plane, arrows), and a Merci stent retriever resulted in successful mTICI IIB reperfusion (H: coronal plane showing distal embolization resulting in new A2 occlusion, I: sagittal plane). MRI, magnetic resonance imaging; MRA, magnetic resonance angiogram; MCA, middle cerebral artery; DSA, digital subtraction angiography​​​​​;​​ ICA, internal carotid artery; CSO, carotid stent occlusion; IA-tPA, intra-arterial tissue plasminogen activator; ET, endovascular treatment, mTICI, modified thrombolysis in cerebral infarction.

## Appendices

Detailed ET of each acute CSO

Patient 1: Left Acute CSO Seven Days Post Stent Placement

A proximal left ICA stent was placed for severe symptomatic stenosis prior to coronary artery bypass surgery. Dual antiplatelet (DAPT) therapy with aspirin 81mg and clopidogrel 75mg daily was started one week prior to stent placement. Seven days after stent placement, the patient developed acute right-sided hemiparesis (NIHSS 4). The patient was evaluated at 0.5 hours after symptom onset, but the patient was not an IV-tPA candidate due to recent cardiac surgery. CT angiography and perfusion (CTA/P) demonstrated acute left CSO, no intracranial large vessel occlusion, a small core infarction, and significant tissue at risk of infarction (Figure 3). The patient underwent ET.

Left common carotid artery access was obtained as described. Next, a 5-Max-Ace catheter (Penumbra, Alameda, CA) was advanced through the thrombosed stent under continuous aspiration and into the more distal cervical left ICA. Moderate resistance to 5-Max-Ace advancement was appreciated during this aspiration thrombectomy. The 5-Max-Ace was removed under continuous aspiration. DSA demonstrated partial stent recanalization with residual non-occlusive thrombus in the mid-section of the stent. Intracranial angiography following aspiration thrombectomy was not performed. Next, an Accunet distal embolic protection device (Cook Medical, Bloomington, IN) was placed in the left ICA distal to the stent. A 4x12mm monorail balloon catheter (Boston Scientific, Marlborough, MA) was advanced over the guidewire into the stent such that it bridged the area of residual thrombus, and angioplasty was performed to macerate the residual thrombus against the wall of the stent. Post angioplasty DSA demonstrated improved stent caliber with a minimal residual internal filling defect. No tandem intracranial occlusion was present. The Accunet was removed, and the procedure was concluded. ET for this patient is shown in Figure 3.

The retrospective chart review demonstrated inconsistent clopidogrel administration. Subsequent platelet mapping studies after therapeutic clopidogrel reloading demonstrated moderate aspirin resistance (arachidonic acid, 59.8% inhibition) and significant clopidogrel resistance (adenosine diphosphate, ADP, 2.8% inhibition). The DAPT regimen was changed to aspirin 81mg and prasugrel 10mg daily, and the patient had no further complications. Three months following ET, the patient’s NIHSS was 0 and mRS score was 1.

Patient 2: Right Acute CSO Eight Hours Post Stent Placement with Tandem Middle Cerebral Artery Occlusion

A right ICA stent was placed for symptomatic stenosis in a patient with atrial fibrillation. The patient’s coumadin was stopped two days prior to stent placement, with subsequent loading with clopidogrel 300mg and maintenance clopidogrel 75mg daily. Following stent placement, procedural heparinization was reversed with 100mg protamine to facilitate groin closure. Eight hours after stent placement, the patient had acute onset of left-sided hemiparesis and neglect (NIHSS 13). Virtual CT angiography (derived from CT perfusion images) revealed tandem occlusions of the right inferior M2 and A2 segments of the middle and anterior cerebral arteries. CT perfusion images were consistent with right CSO, no detectable core infarction, and 222 mL of tissue at risk (Tmax >6 sec, Figure 4). The time from deficit onset to the presentation was 0.25 hours; however, IV-tPA was deferred given multiple lower extremity vascular surgeries in the prior 24 hours. The patient underwent ET.  

Right common carotid artery access was obtained with a 6-French shuttle sheath as described. Aspiration thrombectomy of the CSO was performed with an ACE68 catheter (Penumbra, Alameda, CA). Post-aspiration DSA demonstrated partial stent recanalization without antegrade filling of the cerebral circulation. An attempt was made to place an Emboshield embolic protection device (Abbott, Abbott Park, IL) through the partially occluded stent, but the device could not be navigated through the stent due to resistance. Therefore, the stent was crossed with a Velocity microcatheter (Penumbra, Alameda, CA) over a Traxcess microwire (MicroVention, Tustin, CA) with a docking wire extension (MicroVention, Tustin, CA). The microwire was positioned in the right petrous ICA, and the microcatheter was exchanged for a non-compliant Trek 5x15mm balloon microcatheter (Abbott, Abbott Park, IL). Stepwise angioplasty of the stent was performed. Post-angioplasty DSA demonstrated improved stent and cervical ICA recanalization with residual partially occlusive thrombus in the cervical ICA and tandem right inferior M2 segment occlusion. Repeat stent and cervical ICA angioplasty plus aspiration thrombectomy was performed with subsequent complete recanalization of the cervical vessels.

In order to treat the inferior M2 segment occlusion without repeated crossing of the carotid stent, the ACE68 catheter was again advanced through the carotid stent, and the 6-French shuttle sheath was then advanced over the ACE68 such that it was positioned distal to the carotid stent. The inferior M2 segment occlusion was then treated by combined aspiration and mechanical thrombectomy using a 4x20mm Solitaire (Medtronic, Irvine, CA) and the ACE68. mTICI III reperfusion of the right MCA was achieved after two passes. ET for this patient is shown in Figure 4.

A thorough case review suggested that the most likely precipitant of the patient’s CSO was intravenous protamine administration for heparin reversal in this patient with known atrial fibrillation. Platelet mapping studies demonstrated an appropriate response to aspirin and clopidogrel. The patient was discharged with an NIHSS of 3 on clopidogrel 75mg daily and coumadin for concomitant atrial fibrillation. Three months after ET, the patient was walking independently and had improved to an mRS score of 3. However, the patient subsequently developed heart failure, a urinary tract infection, acute kidney injury, and severe toxic metabolic encephalopathy and died 3.2 months after ET.

Patient 3: Left Acute CSO Six Days Post-Stent Placement with Tandem Anterior Cerebral Artery and Middle Cerebral Artery Occlusions

A left ICA stent was placed for a symptomatic stenosis, and the patient was discharged on aspirin 81mg and clopidogrel 75mg daily. The patient was noncompliant with these antiplatelet medications and presented six days later with acute onset right hemiparesis and aphasia (NIHSS 20). The patient was not an IV-tPA candidate as the time from symptom onset to medical evaluation was 12 hours. MRI demonstrated a small left watershed infarction. MRA showed bilateral ICA occlusions (the right cervical ICA occlusion was chronic) and tandem occlusions of the left inferior M2 and distal left A2 segments (Figure 5).

The patient underwent emergent ET using aspiration thrombectomy with the 5-Max-ACE catheter and 5-Max separator (Penumbra, Alameda, CA). Post-aspiration DSA demonstrated partial stent recanalization with slow antegrade filling. An Accunet distal embolic protection device was placed in the left ICA distal to the stent. Next, a Trek rapid exchange 5x12 mm balloon catheter (Abbott, Abbott Park, IL) was used to perform angioplasty of residual in-stent thrombus. A post angioplasty DSA demonstrated markedly improved caliber of the stent with minimal residual filling defect within the stent.

Following recanalization of the carotid stent, there was now robust antegrade filling of the cerebral circulation, but tandem occlusions of the left temporooccipital artery (distal M2 segment) and left anterior cerebral artery (A2 segment) were still present. Intra-arterial tissue plasminogen activator (IA-tPA) was administered into the left ICA and into the occluded left temporooccipital artery with successful thrombolysis of the left anterior cerebral artery and partial recanalization of the left temporooccipital artery (mTICI IIB).

Post-procedure, the patient was maintained on aspirin 81 mg and clopidogrel 75 mg daily. The patient remained aphasic and with right sided hemiparesis, but able to spontaneously move the left side to commands (NIHSS 18). One month after ET, the patient had a NSTEMI that resulted in cardiopulmonary collapse and severe ventilator associated pneumonia. Comfort care was initiated and the patient expired.

Patient 4: Left acute CSO 18 months post stent placement with anterior cerebral artery and middle cerebral artery occlusions

This patient had prior bilateral carotid endarterectomies followed by left carotid stent placement for a recurrent left ICA stenosis. The patient was maintained on aspirin 325mg and clopidogrel 75mg daily after stent placement. Interval surveillance CTA studies showed no intimal hyperplasia or other complications. 18 months after stent placement, the patient discontinued all antiplatelet agents and developed aphasia and right hemiparesis (NIHSS 21). The patient was not an IV-tPA candidate as the time from symptom onset to medical evaluation was 7 hours. MR perfusion demonstrated 2 ml core infarction in the left frontal lobe and 92 ml of tissue at risk of infarction (Tmax > 6 sec, Figure 6). MRA demonstrated a left ICA occlusion. The patient underwent ET.

DSA demonstrated a left CSO without antegrade filling of the cerebral circulation. The occluded stent was successfully crossed with an Accunet embolic protection device, and angioplasty of the CSO was performed with a Viatrac 6x20mm balloon catheter (Abbott, Abbott Park, IL). DSA following angioplasty resulted in complete stent recanalization.

After angioplasty and complete stent recanalization, DSA subsequently revealed a tandem left carotid terminus occlusion. IA-tPA (5 mg) was administered into the left ICA, which partially dissolved the left ICA terminus thrombus. Occlusion of the left M1 segment and left A1 segment was then appreciated. A residual left M1 occlusion was treated using a Merci retriever (Concentric Medical, Mountain View, CA) with successful recanalization after three passes (mTICI IIB). The residual A1 occlusion spontaneously resolved. However, the post thrombectomy DSA demonstrated contrast extravasation into the basal ganglia, which was consistent with a cerebral reperfusion hemorrhage.

Post-procedure, the patient’s mental status declined, and a follow-up head CT showed a large intraparenchymal hemorrhage. The patient was transitioned to comfort care and expired.
